# Perceptual interventions ameliorate statistical discrimination in learning agents

**DOI:** 10.1073/pnas.2319933121

**Published:** 2025-06-16

**Authors:** Edgar A. Duéñez-Guzmán, Ramona Comanescu, Yiran Mao, Kevin R. McKee, Ben Coppin, Suzanne Sadedin, Silvia Chiappa, Alexander S. Vezhnevets, Michiel A. Bakker, Yoram Bachrach, William Isaac, Karl Tuyls, Joel Z. Leibo

**Affiliations:** ^a^Google DeepMind, Google UK Ltd., London EC4A 3TW, United Kingdom; ^b^Independent researcher, London N1C 4DN, United Kingdom

**Keywords:** statistical discrimination, reinforcement learning, partner choice, perceptual interventions

## Abstract

Choosing social partners is a potentially demanding task which involves paying attention to the right information while disregarding salient but possibly irrelevant features. The resultant trade-off between cost of evaluation and quality of decisions can lead to undesired bias. Information-processing abilities mediate this trade-off, where individuals with higher ability choose better partners leading to higher performance. By altering the salience of features, technology can modulate the effect of information-processing limits, potentially increasing or decreasing undesired biases. Here, we use game theory and multiagent reinforcement learning to investigate how undesired biases emerge, and how a technological layer (in the form of a perceptual intervention) between individuals and their environment can ameliorate such biases. Our results show that a perceptual intervention designed to increase the salience of outcome-relevant features can reduce bias in agents making partner choice decisions. Individuals learning with a perceptual intervention showed less bias due to decreased reliance on features that only spuriously correlate with behavior. Mechanistically, the perceptual intervention effectively increased the information-processing abilities of the individuals. Our results highlight the benefit of using multiagent reinforcement learning to model theoretically grounded social behaviors, particularly when real-world complexity prohibits fully analytical approaches.

## Significance

Undesired bias is a societal problem that is often exacerbated by technology. Faced with limited information and time, people may choose their social partners based on prior experience with people that resemble the potential partner, a behavior called statistical discrimination. We show that machine learning agents are also prone to statistical discrimination and that augmenting their cognitive ability does not solve the problem. However, interventions that highlight relevant information during partner choice can be effective, allowing agents to make choices that are both more rewarding and less biased. We suggest that such perceptual interventions may be a practical way to reduce undesired bias in learning systems, with possible applications to humans.

Choosing good social partners is one key to successful human cooperation (1), but accurately evaluating a potential partner requires acquiring, integrating, and processing information across a variety of situations (2, 3). Information-processing trade-offs create pressure to learn and apply cheap heuristics in partner choice (4). These heuristics, however, can produce undesired biases. Such biases are evident in both human behavior (5, 6) and machine learning algorithms (7, 8), and have clear negative effects on economic efficiency and human well-being (9–14).

Economists have studied the emergence of biases in social decision-making for decades (15–17). One framework is statistical discrimination, which occurs when a decision maker must assess an individual’s suitability as a partner but lacks immediate access to features that are causally relevant to that choice. Instead, the decision maker bases their assessment on features that are readily available and, in their experience, correlate with the ones of interest (16, 18). Statistical discrimination stands in contrast to taste-based discrimination, where decision makers have an intrinsic preference for one group over another (19). Models of statistical discrimination do not attempt to capture the full complexity of racial, gender, and other types of discrimination in human societies, which likely involve a wide range of cognitive phenomena such as in-group favoritism (20), group entitativity (21), and more. Nonetheless, it is possible that multigenerational patterns of systematic social exclusion could be initiated and/or supported by statistical discrimination (22).

The original models of statistical discrimination assumed a decision maker evaluates a referent individual in a one-shot interaction where only perceptible information is available, such as a single job interview (16). Further models introduced imperfect observation of skills (in the form of a worker qualification level) (23). Significant empirical literature has been developed assessing the presence and effects of statistical discrimination in the real world (9, 12, 24–26). However, our understanding of how statistical discrimination is mediated by bounded rationality and learning dynamics is limited, particularly outside of one-shot interactions.

Real-world decision makers can improve their knowledge of the outcome-relevant characteristics of social partners in a gradual manner. Employers in the real world, for example, often refine their assessment of job applicants with successive filters and stages via résumé screening, multiple rounds of interviews, internships, and other provisional employment opportunities that improve their knowledge of the applicant’s suitability for a job. In other words, real-world situations are temporally extended, require acquiring, processing, and integrating information across time, and comprise a complex tapestry of uncertainty, repeated interactions, and risk management. Even with perfect availability of information, a boundedly rational individual might not be able to process the information and act optimally. This is especially likely where social evaluations are rapid; humans make more mistakes when making decisions under time pressure (27), and these results transfer to social interactions (28) including in responses to discrimination (5, 29). Implicit bias is a measurable and unconscious process that biases perception of social partners based on their perceived group membership (5). Reduction of implicit bias is possible but appears to require awareness of the bias, and a conscious effort to diminish it (30) [cf. (31)]. Limits to time, information-processing ability, or the reliability of signals of partner quality will therefore impact the accuracy of decision-making, and produce higher bias.

## Mitigating Bias by Modifying Information Salience.

Since statistical discrimination is a result of constraints on information use, it may be amenable to corrections that address these constraints. Increasing individuals’ information-processing effort or ability is one possible way to achieve this, as seen in studies that reduce implicit bias. For example, Devine et al. (30) designed a multifaceted prejudice habit-breaking intervention targeting the subject’s awareness and concern over their behaviors being biased. But increasing effort or cognition is often not feasible. Alternatively, systems that remove spurious correlations or explicitly increase the salience of outcome-relevant features could ease the information-processing pressure on a learning agent. Perceptual intervention in a partner choice system for humans, for example, could reduce bias without awareness or conscious effort by highlighting outcome-relevant features, or de-emphasizing spuriously correlated ones. Such perceptual interventions might take many forms, from a virtual assistant, to a reputation score, to an augmented reality layer.

Technology plays an increasing role in human decisions, including partner choice. Use of technology has been shown to exacerbate biases in a wide range of partner choice scenarios, including dating (32), ride sharing (33), and online communities (34). However, there is also hope that we could design technologies to help reduce biases, and thus lead us to make better decisions (35–38). For example, virtual reality has been used to reduce bias by explicitly placing individuals in situations that challenge their assumptions, such as showing their avatars as a different race or gender (39–41).

## Reinforcement Learning As a Model of Bounded Rationality.

In reinforcement learning, agents interact with an environment with the goal of maximizing reward (42). The reward is a real-valued signal from the environment that the agent accumulates, analogous to utility in economics, and score in games. In machine learning, agents interact with the environment in discrete time intervals called episodes and use that experience to reinforce actions that lead to higher cumulative reward. Reinforcement learning agents have gained popularity by achieving high performance on many complex tasks, including board games (43, 44), video-games (45, 46), and robotics (47, 48).

Multiagent reinforcement learning has recently emerged as a framework for the study of social interactions (49–52) by generalizing traditional models of social behavior to more realistic scenarios in two important ways. First, it enables study of more complex environments than the ones covered by traditional game theory methods; the environments can be spatial, and temporally extended, grounding the abstract conceptions of actions and interactions in a rich world. Second, it enables the study of agents in those rich environments whose decision processes are limited by learning algorithms and information-processing ability, and whose interactions are grounded by the laws of their worlds.

In contrast to many game theoretic models that assume optimal behavior, models of bounded rationality explore the role of limited information-processing ability and biases (53) in the dynamics and behaviors of a population. Reinforcement learning offers a fresh approach to modeling bounded rationality, focusing on the learning dynamics, where the bounds are implicit to the learning process (51, 54). These tools have been used to study a variety of social situations, including social dilemmas (51, 55, 56), norm and convention formation (57–59), communication (60, 61), and many more. A recent article explored partner choice in an abstract iterated social dilemma environment (62), validating that partner choice can indeed promote cooperation in learning agents.

In this work, we use a combination of multiagent reinforcement learning and analytical modeling to study the learning dynamics of agents engaging in partner choice. Our reinforcement learning agents stand as a model of boundedly rational individuals making decisions under time constraints and through repeated interactions. In-depth exploration of these agents’ behavior in a single complex environment allows us to examine how endogenous learning dynamics differ from the optima predicted in abstract games. This approach parallels longstanding practice in the literature where a single representative problem of interest is explored, for instance in economics (10, 16, 23), and in multiagent reinforcement learning (63, 64). Meanwhile, our analytical model captures the expected dynamics in an iterated game without this environmental complexity and supports the generality of our findings by showing that any environment sharing the same incentive structure (i.e. iterated Stag hunt) would show a similar pattern. We focus on iterated Stag hunt because it is one of the best-studied coordination games and captures many real world scenarios (65).

Together, our simulations and analytical results show that agents engage in statistical discrimination even to the detriment of their overall payoff. Agents with higher information-processing abilities perform slightly better, but remain highly biased. Perceptual interventions that increase salience of outcome-relevant features reduce bias in partner choice. Stronger interventions lead to higher debiasing effects, but even subtle interventions result in reduced bias.

## The Model

**Fig. 1.**
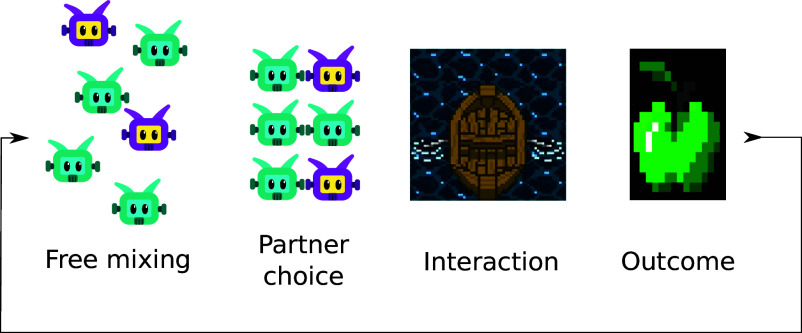
The stages of iterative partner choice: During free mixing, individuals have access to observable features, but not directly their suitability as partners. Individuals form pairs based on what information is available to them and proceed to interact. The outcome of the interaction is a pair of payoffs (or rewards) assigned to the individuals in each of the pairs.

We abstract the scenario of choosing desirable social partners to engage in potentially mutually beneficial interactions as an iterated game. Each iteration consists of several phases (Fig. 1). A free mixing phase where individuals can observe some characteristics of others that might or might not correlate with actual performance. Crucially, the actual partner quality cannot be directly observed in this phase. A partner choice phase where individuals pair up, and commit to interact to the exclusion of all other possible social partners. After choosing partners, the pairs continue to the interaction phase, where the outcome-relevant behaviors are expressed. The outcome phase assigns payoffs (or rewards) to each of the individuals based on how they, and their partner, behaved during the interaction phase. We choose this formulation because it captures a wide range of realistic social situations where partner choice is important and information on partner quality is, at least initially, limited.

### Boat Race Environment.

**Fig. 2.**
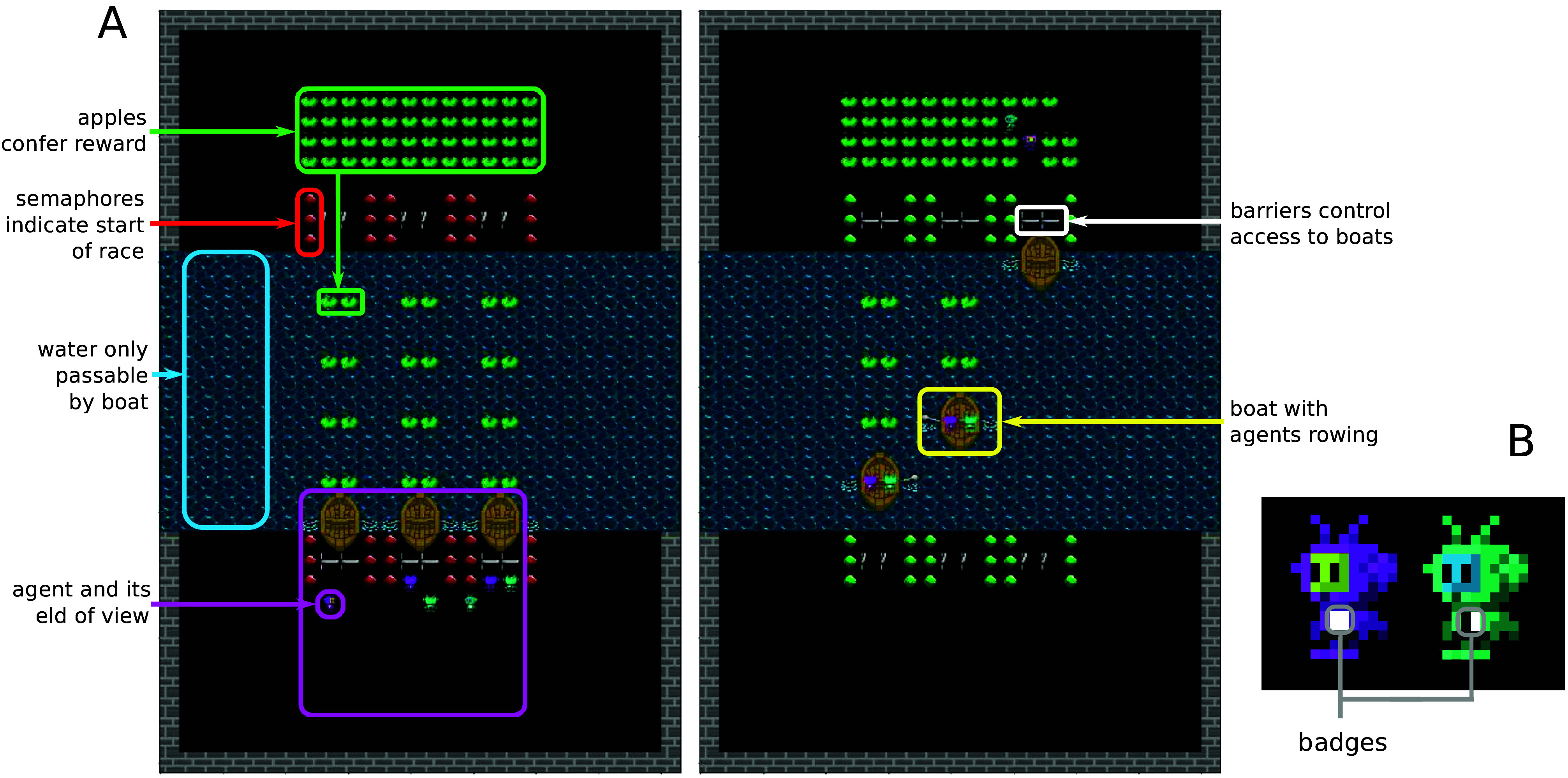
The *boat race* environment. (*A*) Six players (teal and purple) pair up to row boats (brown) and cross the river to access apples (green) that confer rewards. Agents can move freely on the river banks (black) but cannot walk across the river (water sprites in blue). Access to the boats is gated by barriers (gray). On the *Left*, we see a frame from before a race starts (semaphores are red) and agents are able to move freely. On the *Right*, we see a frame from after the race has started. The semaphores have turned green, and the barriers lifted to provide access to the boats. (*B*) Avatars are assigned a unique random “badge” at the beginning of an episode so their identity can be known throughout the episode.

We created a virtual environment that captures the properties of iterated interactions with partner choice. The environment is spatially and temporally extended, and information about the interactions and outcomes of self, partners, and third parties, is perceptual. The environment was inspired by the boat rowing thought experiment posed by Hume (66); see Fig. 2*A*]. 6 players in the environment engage in 8 back-and-forth boat races across a river to reach apples that confer a reward. Boats, however, cannot be rowed by a single player, and thus, players need to find a partner before each race and coordinate their rowing during the race to cross the river. We validate that our results are not dependent on the specific implementation of an environment by creating a theoretical model of iterative partner choice (*Materials and Methods*).

The dynamics of the interactions are chosen to correspond to an iterated version of the Stag hunt game (67), which was extensively studied by Skyrms (68). The traditional Stag hunt game captures the incentive structure for a pair of coordinating individuals. There is a higher reward to be had if they both hunt the stag, but there is an inherent risk in this decision: attempting to hunt the stag alone results in injury, whereas a safe option of hunting hare is always available. We chose Stag hunt instead of the Prisoner’s dilemma as the underlying dynamics of the social interactions because it captures situations where there is mutual benefit to be had, without a temptation of betrayal. In contrast to the Prisoner’s dilemma where there is always an incentive to defect, the incentive in Stag hunt is to coordinate with your partner, albeit with risk. Skyrms argued that because of these properties, Stag hunt captures the dynamics of long-term cooperation better than the Prisoner’s Dilemma, and thus is the most likely basis of social contract formation in humans (68). Skyrms showed that partner choice and signal evolution (even given unreliable signals) can stabilize partner choice (and thus, cooperation) via the emergence of social networks. Yet the dynamics of partner choice in complex multiagent settings remain poorly understood. In our environment, the two strategies are captured by the way players row the boat: paddling, the coordinated but risky option which corresponds to playing “stag” in Stag hunt and flailing, the safe but low reward option corresponding to playing “hare.”

At the beginning of each race, access to the boats is blocked by barriers. The barriers lift after a predetermined amount of time, giving the players time to move freely and approach the boat or partner of their choice (the “free mixing” phase; see Fig. 1). The topology of the environment is such that once a pair of players is behind the barriers of a boat, no other player can force themselves into that boat (this corresponds to the “partner choice” phase). If a player so wishes, however, they can back away and walk to another boat. Once a player reaches a seat, they remain seated until either they reach the other river bank, or the time allotted for the race is up, in which case they are disqualified and removed for the rest of the episode. When a boat has a player on each seat, it can be rowed as described above (the “interaction” phase). The environment contains apples that provide reward to the player that touches them. The apples are consumed in the process, but will be replenished eventually (see below). They are positioned in two different locations: along the river to encourage progress; and on the opposite river bank from the players, as a reward for finishing the race (the “outcome” phase).

The apples on the river are replenished at the start of each race. The apples on the river bank replenish at a constant rate, but disappear altogether when the race time is up (and reappear on the opposite bank). The replenishing rate of the apples on the river bank is high enough that even ALL players optimally consuming them cannot exhaust them. There is a strong incentive to finish a race quickly, so as to accrue the largest reward. For full details, see *SI Appendix*.

Two perceptible attributes are given to distinguish players (Fig. 2*B*). First, every player is colored purple or teal. The color is readily observable as the players perceive their environment as an image window around themselves (Fig. 2*A*), but is insufficient to individualize a player’s behavior. Second, a badge, is given to every player at the start of the episode, which uniquely identifies them within the episode. The badge is a pattern of 2×2 dark gray or white pixels at the center of the player’s sprite. The badge is not persistent across episodes.

### Experimental Setup.

Our experiments are structured in two parts: training and evaluation.

#### Training.

We train a focal agent in a community of other agents. This community is called the training community. Agents in the training community are incentivized to either always paddle (cooperate) or flail (defect) when in a boat (half of each; see *SI Appendix*). We control the correlation between player color and their rowing style using a parameter that we refer to as training bias or color-strategy correlation. The training community will always have half its agents of each color. The focal agent is trained in episodes that randomly sample 5 agents from the training community for each episode. This means that on average, the focal agent will be on episodes where half the partners are of each color and also half of the partners are of each strategy, but color and strategy are (spuriously) correlated.

#### Evaluation.

After training for $10^{9}$ environment steps, we freeze the focal agent’s parameters and expose them to a new community where there is no correlation between player color and strategy. We call this unbiased community the evaluation community (see *SI Appendix* for more details). We measure the behavior of the focal agents in the unbiased evaluation community. This setup mimics the situation where individuals growing up might encounter a skewed distribution of social partners that need not be reflective of the population at large. Reaching adulthood, however, they might encounter social partners without that skew. We want to understand whether individuals growing up in a biased environment would carry that bias even in situations where the bias would not only be outdated but disadvantageous.

### The Discrimination Index.

To measure the degree of statistical discrimination exhibited by focal agents, we keep track of the partner choices made during the multiple races in an episode and aggregate them across episodes. Since we know the ground truth of the strategies of agents in the training community, we only need to keep track of associations with cooperators and with defectors of either color. The resulting summary is called an association matrix. The association matrix $P^{\left(i\right)}$ at a particular iteration (race) *i* is a 2×2 matrix of proportions where $P_{j}^{\left(i\right)}$ corresponds to the proportion of times the focal individual shared a boat with a partner with color ∈{p,t} (for purple and teal, respectively) and strategy ∈{c,d} (for cooperator and defector, respectively). For instance, $P_{t,d}^{\left(i\right)}$ corresponds to the proportion of times the focal individual associated with a teal defector. When the iteration is clear from context, or irrelevant, we will drop the superindex and simply use *P*.

We define the discrimination index of a focal individual at iteration *i* as

$$D_{i}=\left|P_{p,c}^{\left(i\right)}-P_{p,d}^{\left(i\right)}\right|+\left|P_{t,c}^{\left(i\right)}-P_{t,d}^{\left(i\right)}\right|-\left|P_{p,c}^{\left(i\right)}-P_{t,c}^{\left(i\right)}\right|-\left|P_{p,d}^{\left(i\right)}-P_{t,d}^{\left(i\right)}\right|$$

This index measures the difference between how much an agent is associating based on partner behavior: $\left|P_{p,c}-P_{p,d}\right|+\left|P_{t,c}-P_{t,d}\right|$ (sum of absolute differences across columns); versus how much it is associating based on partner color: $\left|P_{p,c}-P_{t,c}\right|+\left|P_{p,d}-P_{t,d}\right|$ (sum of absolute differences across rows). A negative value means the agent prefers to associate with bots based on their color rather than their behavior. A positive value means they prefer to associate by behavior rather than color. A value of zero means that they oversample a color, only insofar as they oversample a behavior (i.e. there isn’t a row or a column that is larger element-wise than the other; see *SI Appendix*). The discrimination index is able to detect that an agent is engaging in statistical discrimination, because if they associate with a particular color disproportionately, D<0.

An optimal rational decision maker would show statistical discrimination in the first iteration ($D_{0}<0$), but would not in the last iteration ($D_{7}>0$). The reason being that at the beginning of an episode, the focal agent has no better information than the partners’ colors to decide. The optimal choice for partners is one of the color that has the higher correlation with cooperation, leading to negative $D_{0}$. However, after sampling various partners, the focal agent should attempt to partner exclusively with known cooperators (ignoring color), leading to positive $D_{7}$.

### Experimental Conditions.

One focal agent is trained in a training community with each color-strategy correlation. For a particular experimental condition, we train independent focal agents of each color, and on a range of training biases. The baseline agents have the ACB (actor-critic baseline) architecture (69, 70), composed of a convolutional neural network, followed by a feed-forward, fully connected network, feeding into a Long-Short-Term-Memory module, from which the policy and value estimation heads are computed (more details in *SI Appendix*).

We compare the baseline agent with an agent with the same overall architecture but without a memory module. This is intended to capture the difference between automatic, heuristic decision-making (no memory), and more deliberate, careful consideration by individuals (with memory). It also serves to directly control for the information-processing abilities of the agent.

To measure the effects of perceptual interventions, we train focal agents with the baseline architecture, each with different intensities of perceptual interventions. The focal individual sees a crown on players who largely cooperated (paddling at least 75% of their rowing) during the last interaction (race). The crown can only appear during the interaction phase and lasts until it decays or the next partner choice phase is reached, whichever is sooner. The salience of this intervention is controlled by a decay parameter *β*. We set *β* to values that would cause a perfect cooperator’s crown to disappear soon after the end of the race at the beginning of the outcome phase (0.02), half way through the outcome phase (0.005), half way through the free mixing phase (0.002), and until the next partner choice phase (0.0) (*Materials and Methods* and *SI Appendix*). The higher the decay, the sooner the crown disappears and thus the higher information processing (through memory) the focal agent must possess to effectively use this signal. Crowns are an honest and perfect signal of partner quality.

## Results

We measure the first ($D_{0}$) and last race ($D_{7}$) discrimination index of each focal agent (for each training bias) across 600 episodes with the (unbiased) evaluation community. The focal agents are not allowed to learn using gradient updates from these test episodes.

**Fig. 3.**
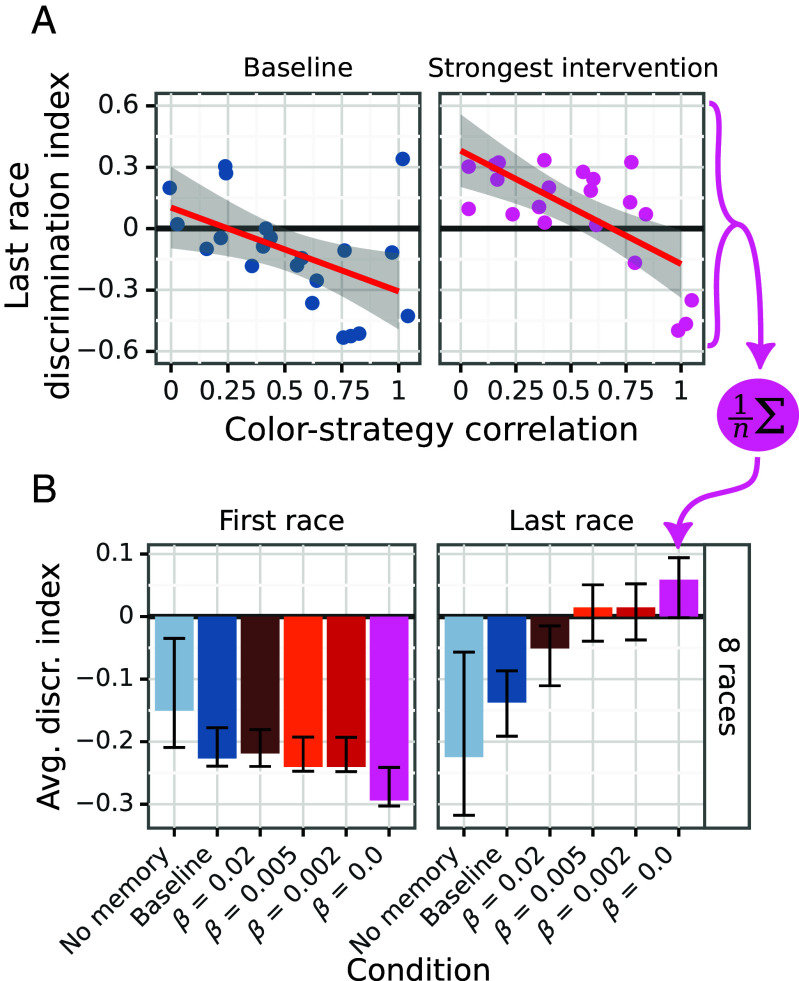
Focal agent’s discrimination index D in their evaluation communities. Positive values indicate choosing partners based on their behavior, negative ones indicate choosing based on their color. (*A*) $D_{7}$ in evaluation communities as a function of training bias. The higher the training bias, the worse the discrimination index is. On the *Left*, the baseline shows negative $D_{7}$ overall. On the *Right*, the strongest perceptual intervention shows positive $D_{7}$ overall. (*B*) D averaged over training biases is shown for the experimental conditions: no memory, baseline, and four levels of decay *β* of the perceptual intervention. Stronger perceptual interventions (those with less decay) result in improved $D_{7}$. CIs are computed by bootstrapping each agent’s discrimination index (5,000 samples).

Focal agents trained without perceptual interventions engage in statistical discrimination (i.e. exhibit negative discrimination index; see Fig. 3). They rely on partner color to make decisions about who to associate with. This pattern persists across iterations (races) within an episode, despite this being a suboptimal strategy in terms of rationality and payoff (Fig. 3*B*). Focal agents trained in highly biased communities tend to exhibit worse discrimination at test time. The larger the training bias, the more negative their discrimination index at test time.

Interestingly, the relationship between training bias and the discrimination index is roughly linear (Fig. 3*A* and *SI Appendix*). This is surprising because rational utility optimizers should maximally exploit any detectable bias, which would result in a threshold response to increases in training bias.

**Fig. 4.**
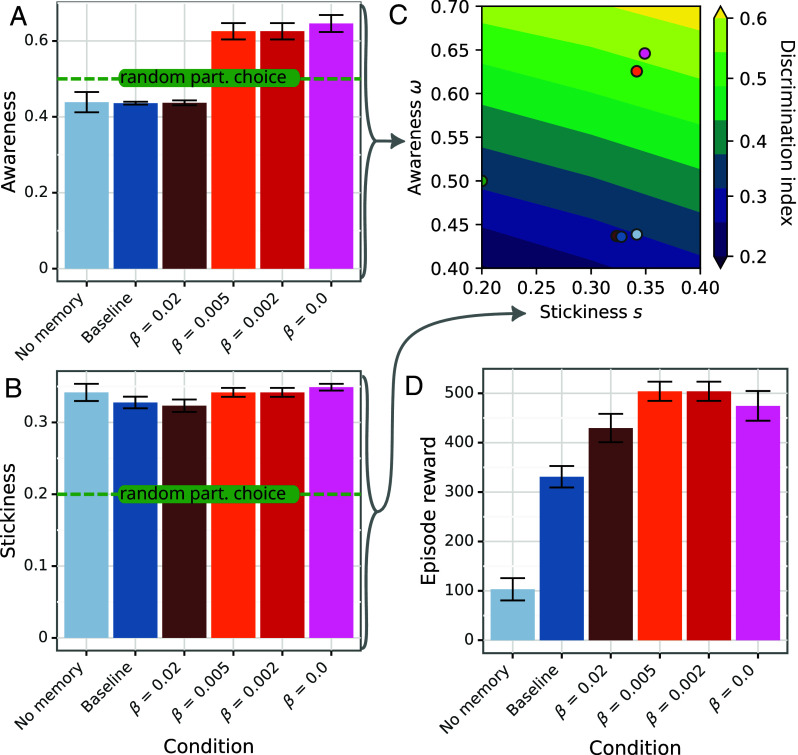
(*A* and *B*) The empirical values of awareness and stickiness for the focal agents, respectively. (*C*) The discrimination index for the analytical model, computed from $P_{j}^{\left(8\right)}$. Each figure shows a contour plot of the average discrimination index across all color biases (*B*) in terms of the stickiness *s* (on the *x*-axis) and awareness *ω* (on the *y*-axis). The empirical stickiness and awareness of the baseline agents and those trained with perceptual interventions are shown overlaid on the graph. (*D*) The average episode reward of the focal agents. Error bars correspond to SE.

Agents without a memory perform worst in terms of their discrimination index. Lacking memory means it is virtually impossible for them to improve their discrimination index in successive races, as they are unable to identify good partners to choose for the next interaction (Fig. 3*B*). Agents without a memory also receive less reward on average (Fig. 4*D*).

Perceptual interventions ameliorate or reverse the baseline discrimination on the last race, depending on the strength of the intervention (see Fig. 3, on the *Right*). The stronger the perceptual intervention, the better the average discrimination index is at test time (Fig. 3*B*, *Right* panel). Better discrimination index translates to better reward performance, showing that perfect rationality favors association by color and ignoring the spurious correlation (Fig. 4*D*). The strongest intervention (*β* = 0.0) makes the last race discrimination index positive on average for all training biases except the extreme of 1 (Fig. 3*A*, *Right* panel). For training bias 1, there is no extra information given by the perceptual intervention because there is already a perfect correlation between color and behavior. Because perceptual interventions are only triggered during an interaction, they have virtually no impact on the discrimination index on the first race, as expected (Fig. 3*B*, *Left* panel).

### Analytical Model of Iterated Partner Choice.

To better understand the effects of perceptual interventions on a focal individual facing iterated partner choices, we develop an analytical model. The model calculates the discrimination index exhibited by a focal individual with varying information-processing abilities. Potential partners can be of either purple or teal color, and either good social partners or bad ones.

We characterize the focal individual by three parameters:

- Awareness (*ω*): The ability to identify and partner with good social partners by observing their interactions with others.
- Bias (*b*): The bias in their default preference to associate with purple coplayers.
- Stickiness (*s*): The ability to partner again with a social partner with whom they had a good interaction in the immediately previous iteration.

We use these three parameters because they capture the essence of information-processing abilities and intrinsic bias, and because they can be empirically estimated by observed behavior (including that of reinforcement learning agents). See Table 1 for a description of all the parameters of the model.

**Table 1.** The parameters of the analytical model

Parameters	
Individual	Description
*s*	stickiness: the probability that if they like their partner, they re-pair on the next iteration.
*ω*	awareness: the probability that if they must choose a new partner, they pair with a cooperator, regardless of color.
*b*	bias for purple partners, absent stickiness or awareness.
J	Set of partner types {(p,c),(p,d),(t,c),(t,d)}.
$P_{j}^{\left(i\right)}$	probability of choosing a partner of type j∈J at iteration *i*; $P^{\left(i\right)}$ denotes the association matrix ${\left(P_{j}^{\left(i\right)}\right)}_{j}$.
*ν*_*j*_	probability that, when choosing a new partner after the first iteration, that partner is of type *j*; *ν* is the vector ${\left(\nu_{j}\right)}_{j}$.
$\gamma^{\left(i\right)}$	probability of choosing a cooperator at iteration *i* (i.e. $P_{\left(p,c\right)}^{\left(i\right)}+P_{\left(t,c\right)}^{\left(i\right)}$ or $1-P_{\left(p,d\right)}^{\left(i\right)}-P_{\left(t,d\right)}^{\left(i\right)}$.)
Population	Description
*ρ*	proportion of purple cooperators
*τ*	proportion of teal cooperators

Empirically, the awareness of a focal individual is the proportion of races in which they picked a cooperator. The stickiness is the proportion of races in which they associated with the same partner in the immediate next race, and the bias is simply the community bias they were trained on.

We calculated the probability that the focal individual chooses a partner that is of either of the four types: purple or teal cooperator; purple or teal defector. This yields the association matrix *P* as a function of *ω*, *s*, and *b*, from which we compute the discrimination index, as in the reinforcement learning case (*Materials and Methods*).

Fig. 4*C* shows the values of the discrimination index for different values of *ω* and *s*, when averaged over *b*. Stickiness only has a strong effect when *s* is close to 1, because convergence of $P^{\left(i\right)}$ is geometric like $s^{i}$, whereas the response to awareness *ω* is linear (see full derivations in *Materials and Methods*.) Intuitively, if an individual is unable to stick with desirable partners resulting in having to sample new partners frequently, there is not much that can be done to be unbiased, other than having high awareness. In addition, having too high stickiness is detrimental to discrimination. This is because sticking to a desirable partner with high fidelity means sticking with an initial (and likely biased) choice, rather than sampling other partners that do not conform to the initial (biased) color preferences. This is exacerbated when awareness is high and there are many iterations.

Fig. 4 *A* and *B* show the empirical awareness and stickiness of the agents in the last race for all conditions: no memory, baseline, and various perceptual intervention decays *β*. These values are also overlaid in Fig. 4*C* to show them in context.

The perceptual interventions effectively increase the awareness of the agent. The response is reminiscent of a threshold effect, where baseline, no memory, and weak interventions result in low awareness, but stronger interventions lead to high awareness. For comparison, an individual sampling completely at random would have an awareness of 0.5 as half the partners are cooperators and half are defectors on average (Fig. 4 *A* and *B*). The no memory, baseline, and weak perceptual intervention agents have an awareness below this threshold. This is most likely because cooperators have already paired up preferentially with each other and any remaining partner will likely be a defector, which leads to awareness being empirically measured as lower. Another explanation for below random awareness is for an agent to harbor a preference for defectors. The data as presented cannot distinguish between these two cases, but we expect a preference for defectors to be highly unlikely.

Perceptual interventions have virtually no effect on stickiness. Choosing random partners would lead to a stickiness of 0.2 (one in five partners are the previous partner). Focal agents show higher stickiness than random, choosing their partner from the last race roughly one third of the time. This effect is likely driven by disqualification, which reduces the partner pool. We see no evidence that this is driven by preference for specific individuals.

## Discussion

We find that reinforcement learning agents readily engage in statistical discrimination. In the absence of information about partner quality, they preferentially associate with partners with perceptible features that correlate with quality. However, reinforcement learning agents do not act as optimal reward maximizers. Most of the tested agents were unable to choose partners based on their strategy, even after this information was repeatedly exposed in interactions.

Optimal behavior in the scenario studied here is well understood via game theory: The game is an iterated, finite-horizon, social dilemma. In our analytical model, we assume agents are maximizing utility with their information-processing availability (awareness) and ability to enact their partner preferences (stickiness). The presented results assume optimal behavior (i.e., a Nash equilibrium for given awareness and stickiness). For the reinforcement learning agents, we can assert that the solutions arrived in the absence of perceptual interventions (or perfect spurious correlation) are not Nash equilibria. Individuals that choose partners in subsequent races in accordance to behavior will achieve higher reward than those selecting them on color.

But why would a learning agent that is trained to maximize reward not achieve that goal? The reason is two-fold. First, the optimal policy is hard to discover and refine. It involves gathering information about partner quality as efficiently as possible, ideally without first-hand experience with them as partners. This is possible in our environment, but a hard perceptual task. An optimal agent would partner with their preferred color, and during the race attend to the pattern of rowing of not only its partner, but of all the players in the episode. Then the agent would memorize the badges of good partners and choose them in subsequent races. Second, the policy enacting simple statistical discrimination is self-reinforcing: Once the agent has learned that on average it is good to partner with one particular color (because there is a high correlation between that color and partner quality), most of its experience would be with partners of that color, confirming and strengthening that policy. Random deviations from that policy would lead to lower expected reward, discouraging exploration. In this sense, our agents are exhibiting a form of confirmation bias (71). Our results show that this canalization of learning (i.e. the inability to disprove the initial bias through experience) is sufficient to make the reinforcement learning agents not behave as rational optimizers.

Both reasons for suboptimality are intrinsic to the learning process of the agent. Modeling such processes with pure analytical approaches can often only be done with ad hoc parameters capturing them. This is also the case in our analytical model, where agent awareness is just an exogenous parameter of the model. However, our reinforcement learning model shows that perceptual interventions can lead to debiasing of agent behavior. We can then theoretically map such interventions to increasing awareness.

### Without Perceptual Intervention.

Statistical discrimination exhibited by our agents intensified when they were unable to obtain, process, or incorporate information on the quality of their social partners. We found that agents trained in a biased community were unable to avoid statistical discrimination on their own. This was expected for agents without a memory module, for whom reciprocating strategies are likely inaccessible. Surprisingly, it was also true for agents with memory. This discrimination was not due to an inability to enact partner choices, since agents trained in extremely biased communities (where color and strategy are perfectly correlated) exhibited a discrimination index at test time that was near the theoretical optimum (*SI Appendix*).

In our setting, theory predicts that small decreases in training bias should have no effect on the policy learned by focal agents. Individuals will still have an incentive to sample the color that correlates highest with cooperation, when available. Interestingly, our agents do not conform to that prediction. They instead respond to small reductions in bias by learning a proportionally less discriminatory policy. This may be due to the stochasticity of sampling coplayers for an episode from a community, which results in a bet-hedging solution on the part of the agent. Essentially, whether an agent uses a signal depends on its reliability, and so as the signal becomes more reliable, it becomes more exploitable. This pattern is compatible with the phenomenon observed in the field of machine learning fairness where reducing bias in the training data has been observed to have a positive impact in the fairness metrics of classification algorithms (72). It is also compatible with the contact hypothesis which postulates that stereotype–disconfirming interactions reduce prejudice in humans (73, 74).

Our results may also be understood within a dual process framework, which posits that slow and deliberative processes may override automatic habit-like processes. Dual process models have a long history in psychology and neuroscience (reviewed in ref. 75). For example, in human vision, feed-forward decision-making operates rapidly (5, 76) and may learn dedicated threat-related features (77), whereas deliberative processes such as mental rotation unfold sequentially via recurrent processing (78), and are consequently much slower (79). These differences parallel the contrast between our purely feed-forward agents (no memory) and ones with recurrent structures (baseline).

A related comparison arises between model-based (MB) and model-free (MF) reinforcement learning (80). Whereas MB learning can prospectively plan to achieve desired outcomes using knowledge of the likely consequences of actions, MF learning gradually updates cached values of actions retrospectively from experience (81, 82). When outcome values change, model-based decision-makers can rapidly change their behavior, whereas model-free decision-makers need additional repetitions (83). In the human brain, MF learning (for example, of habits and rituals) is associated with the dorsolateral striatum (84) while MB learning is associated with persistent activity in the prefrontal cortex (80, 85). By allowing agents to learn relevant features from experience when the task demands it, memory may offer a model for the neural mechanisms that underlie MB learning (85). In our model, agents with memory benefited from incorporating individual-oriented information for partner choice, whereas feed-forward networks (no memory) were incapable of this and thus exhibited worse discrimination. These observations suggest that engaging different neurological systems may modulate the impact of statistical discrimination.

### Perceptual Interventions.

For our agents, perceptual interventions highlighting causally relevant information were highly effective in reducing statistical discrimination. This result contrasts with the above finding that increasing information-processing capacity via memory had little impact on statistical discrimination. Whereas increasing information-processing capacity often demands increased resources, perceptual interventions only require altering the representation of data received by an agent. In this sense, perceptual interventions may represent “low-hanging fruit” for ameliorating statistical discrimination.

The empirical awareness of the agents subject to perceptual interventions exhibited a threshold effect, where weak or no intervention resulted in low awareness, and stronger interventions resulted in high awareness (Fig. 4*A*). We postulate that the intervention is overcoming an information-processing limitation of the agent, and once surpassed, the agent is able to effectively use this information to choose partners.

Much work on AI fairness has focused on the need to debias training datasets. This presents an on-going challenge due to the volume of data and multifaceted and often indirect associations involved in real-world statistical discrimination. Our results show that processing data to increase the salience of causally relevant factors allows agents to overcome biases in their training data, resulting in better performance and (from the perspective of social discrimination) fairer choices. Since this can be done during deployment rather than requiring retraining the model completely, it offers a potentially low-cost technique to address undesired bias. In addition, by altering the processing of a (generally small) number of highly relevant features rather than that of a (potentially very large) number of correlated but irrelevant ones, such perceptual interventions may be relatively simple to implement.

In this work, we used exogenous and perfectly reliable perceptual interventions. This is done to provide the upper limit of what the intervention can achieve. Implementing such an intervention in the real world or for human consumption would require careful consideration and significant oversight, as there would be potential negative effects if the signal has low accuracy, or worse, if it reinforces an undesired bias or is controlled by a malicious actor.

While we focus here on desirable applications of perceptual interventions, another reason to study them is to guard against potential abuse. Perceptual interventions are already used in many contexts to manipulate human decision makers (for example in advertisements and propaganda). Our results highlight that artificial agents are not immune to these manipulations, indicating a need for research into safeguards that can protect both natural and artificial agents. Perceptual interventions that are shared by many agents or people can potentially be highly scalable, giving them dangerous as well as useful leverage, however, they also create a single auditing point that could support enhanced transparency.

An alternative to (exogenous) perceptual interventions is to design agents that are directly better at estimating the quality of others. Framed like this, the partner choice problem becomes that of reputation estimation. There is ample theoretical and empirical literature showing that reputation systems are able to improve cooperation (86, 87), including in reinforcement learning agents (88, 89). This approach is of limited applicability to humans, since it requires augmenting human cognition in an infeasible or unscalable manner.

### Insights.

Our work suggests three general ways to reduce discrimination. The first, widely studied in machine learning, is to decrease the bias in the training community. While desirable (and effective, as our results show), this is challenging and costly due to the complexity and ubiquity of these biases. A second way is through more careful evaluation of potential partners. This requires increased resources and our results suggest it may have limited impact. The third way is through perceptual interventions that increase the salience of causally relevant information upon deployment, an approach that may be cheap, auditable, and scalable. Our results suggest it is also highly effective.

Biased partner choice in humans is likely driven by many complex factors, such as in-group favoritism (20) and beliefs of group trustworthiness (90). However, it is useful to disentangle the effects that specific factors can theoretically have on those choices. Our work suggests that community, perception, and information-processing abilities are intimately involved in strategic decisions of partner choice. Assuming our results generalize to human learners, these observations also have consequences for the amelioration of bias in humans. It is possible to decrease the bias in the environment people grow up with (e.g. via better education, desegregation, antidiscrimination policies, etc.), and this has led to increased social well-being and economic prosperity (91, 92). However, such interventions require not only awareness of the bias but also political consensus at least at a local scale. Increasing a person’s information processing is possible by forcing a deliberate decision instead of a gut reaction, and it also reduces bias (5, 29). However, this mental effort requires attention and time (93). In contrast, perceptual interventions may be a cheaper way to reduce bias, and in our model, they produced a much larger impact on discrimination than either reducing community bias and or adding memory. We already routinely use technology to filter information and make decisions about potential social partners. Reputation systems, for example, are ubiquitous in the internet, and have been presented as a solution to community bias, yet in practice can perpetuate preexisting biases (94). Perceptual interventions do not require consensus at the policy level (other than for regulation and to ensure no unlawful discrimination is occurring), nor do they require knowing a priori which features spuriously correlate with outcome-relevant features, as they can focus on outcome-relevant features directly, bypassing the spurious correlation altogether. Perceptual interventions implemented in reputation systems could be achieved simply with changes to the presentation of information at the user interface, and lead to decisions that are both rationally preferred by users and less statistically biased. Other potential areas that could use perceptual interventions include resume processing systems, social networking sites, and legal and judicial systems.

Discrimination, in all its forms, is a key societal issue. Only by disentangling the factors contributing to its emergence, establishment, and maintenance can we hope to solve this problem. This work contributes to the understanding of the emergence of bias and serves as evidence that using reinforcement learning systems in combination with traditional analytical tools can provide insights into important societal issues.

## Materials and Methods

### Code and Data.

The data for the Figures as well as the code for generating them and configuration of the environment are available at https://github.com/google-deepmind/statistical_discrimination.

### Environment Validation.

We verify that our environment has the properties of an iterated Stag hunt, by computing its Schelling diagram (95, 96) (*SI Appendix*).

### Analytical Model Derivations.

As is typical in iterated social dilemmas, we model interactions as instantaneous. In an interaction, each individual has access to two strategies: cooperate or defect. Individuals then simultaneously choose one strategy and receive a payoff depending on their choice and that of their partner. The payoffs of interactions are those of a Stag hunt game (65, 67),

[1] $$\begin{array}{c} \begin{array}{cc} & \begin{array}{cc} C & D \end{array}\\ \begin{array}{c} C\\ D \end{array} & \left(\begin{array}{cc} R & S\\ T & P \end{array}\right) \end{array} \end{array}$$

where R>T≥P>S.

We consider individuals that can have one of two colors: purple or teal. For simplicity, we will assume that nonfocal individuals will either unconditionally cooperate or unconditionally defect. Let *ρ* be the proportion of purple agents that are cooperators, and *τ* the proportion of teal agents that are cooperators. See Table 1 for a description of the population parameters.

We assume that if the focal individual was happy with the outcome of their previous interaction (e.g. because their partner cooperated), they will succeed in pairing up again with their current partner with probability *s*. We call this probability of pairing the stickiness of the focal individual. Individuals can choose to unilaterally end a relationship by simply choosing someone else. In addition to observing the outcome of the interaction with their partner, individuals might be able to identify good social partners regardless of whether they have direct experience with them. We call awareness the ability of the focal individual to know who the cooperators are, and it is controlled by a parameter *ω*. The combination of stickiness and awareness is how we model information-processing ability in our analytical model. In the reinforcement learning model, this ability to perceive and process outcomes of interactions between other individuals is grounded on the observations of the environment and can be computed from trajectories. Finally, the focal individual has an intrinsic bias toward choosing a particular color. We denote by *b* the probability that the focal individual will partner with a purple individual whenever they fail to stick to their previous partner and are unaware of who the cooperators are (i.e. on the first iteration, or when failed their stickiness and awareness attempts). See Table 1 for a description of the individual parameters.

Let $P_{j}^{\left(i\right)}$ be the probability that at iteration *i*, the focal individual partners with an individual of type *j*. The possible types of partners are j∈J:={(p,c),(p,d),(t,c),(t,d)}, where (p,c) are purple cooperators, (p,d) are purple defectors, etc. Let $\gamma^{\left(i\right)}=P_{\left(p,c\right)}^{\left(i\right)}+P_{\left(t,c\right)}^{\left(i\right)}=1-P_{\left(p,d\right)}^{\left(i\right)}-P_{\left(t,d\right)}^{\left(i\right)}$ be the probability that the focal individual pairs up with a cooperator at iteration *i*.

When the focal individual is forced to choose a new partner after the first iteration (i.e. because their partner defected, or because they failed to stick with them) it will choose a cooperator if it succeeds an awareness test; otherwise, it will choose based on color preference. The vector *ν* denotes the probabilities of choosing new partners. *ν* is composed of the contribution when choosing cooperators, and the contribution when choosing by color. When choosing a cooperator, the probability of choosing a particular color depends only on the prevalence of cooperation by color; this component’s contribution is

$$\begin{array}{c} C:={\left(\frac{\rho }{\rho +\tau },\frac{\tau }{\rho +\tau },0,0\right)}^{T}. \end{array}$$

When choosing based on color the probabilities depend on the bias and the cooperation prevalence by color; this component’s contribution is the same as the first choice $P^{\left(0\right)}$ and is

$$\begin{array}{c} P^{\left(0\right)}={\left(b\rho ,b(1-\rho ),(1-b)\tau ,(1-b)(1-\tau )\right)}^{T}. \end{array}$$

Combining these two components results in the expression

$$\begin{array}{c} \nu =\omega \cdot C+\left(1-\omega \right)\cdot P^{\left(0\right)}. \end{array}$$

$P^{\left(i\right)}$ is then defined recursively as:

[2] $$\begin{array}{c} \begin{array}{cc} P^{\left(i+1\right)} & =\left(1-\gamma^{\left(i\right)}\right)\cdot \nu +\gamma^{\left(i\right)}\cdot \left(s\cdot g^{\left(i\right)}+\left(1-s\right)\cdot \nu \right)\\ & =\left(1-s\gamma^{\left(i\right)}\right)\cdot \nu +s\gamma^{\left(i\right)}\cdot g^{\left(i\right)}, \end{array} \end{array}$$

where

$$\begin{array}{c} g^{\left(i\right)}=\frac{1}{\gamma^{\left(i\right)}}\cdot {\left(P_{\left(p,c\right)}^{\left(i\right)},P_{\left(t,c\right)}^{\left(i\right)},0,0\right)}^{T} \end{array}$$

is the probability the focal individual samples partners (by color) given it is sampling cooperators. In other words, $g^{\left(i\right)}$ is the color distribution of cooperator partners sampled at iteration *i*.

It is possible to derive a closed formula for $\gamma^{\left(i\right)}$ by noticing that in Eq. **2** the only term that contributes the defector sampling probabilities $P_{\left(p,d\right)}^{\left(i\right)},P_{\left(t,d\right)}^{\left(i\right)}$ is the $P^{\left(0\right)}$ term inside *ν*. In particular,

[3] $$\begin{array}{c} \begin{array}{cc} \gamma^{\left(i+1\right)} & =1-P_{\left(p,d\right)}^{\left(i+1\right)}-P_{\left(t,d\right)}^{\left(i+1\right)}\\ & =1-\left(1-s\gamma^{\left(i\right)}\right)\left(1-\omega \right)\left(P_{\left(p,d\right)}^{\left(0\right)}+P_{\left(t,d\right)}^{\left(0\right)}\right). \end{array} \end{array}$$

Eq. **3** is an affine, first-order, autonomous difference equation, with solution

$$\begin{array}{c} \gamma^{\left(i\right)}=e\cdot \frac{1-f^{i}}{1-f}+f^{i}\cdot \gamma_{0}, \end{array}$$

where $e=1-\left(1-\omega \right)\left(1-\gamma_{0}\right)$ and $f=s\left(1-\omega \right)\left(1-\gamma_{0}\right)$. Since 0≤f≤1, as the iteration number increases, $\gamma^{\left(i\right)}$ converges geometrically from *γ*_0_ to $\frac{e}{1-f}$.

Because $\gamma^{\left(i\right)}$ has a closed form formula, we can also express $P^{\left(i\right)}$ as an affine, first-order, difference equation, although it is nonautonomous because it depends on $\gamma^{\left(l\right)}$ for *l* ≤ *i*. After some manipulation, we arrive at the formula for the association matrix

$$\begin{array}{c} P_{j}^{\left(i\right)}=\nu_{j}\left[1+\lambda_{j}\left(\frac{1-s^{i}}{1-s}-1\right)-\sigma_{i}\right]+m_{j}^{i}\cdot P_{j}^{\left(0\right)}, \end{array}$$

where *σ*_*i*_ is an expression depending on sums of $\gamma^{\left(l\right)}$, and where $m_{j}=s$ for cooperators, and $m_{j}=0$ for defectors.

Note that $P_{j}^{\left(i\right)}$ converges geometrically from $P^{\left(0\right)}$ to

$$\begin{array}{c} \nu_{j}\left[1+\left(\lambda_{j}-\frac{e}{1-f}\right)\left(\frac{s}{1-s}\right)\right]. \end{array}$$

We use these formulae to calculate the discrimination index of the focal individual.

## Supplementary Material

Appendix 01 (PDF)

## Data Availability

Pandas Dataframe have been deposited in statistical_discrimination (https://storage.googleapis.com/statistical_discrimination/data/summary_df.feather) (97).
