# Supplementary material for: Perceptual interventions ameliorate statistical discrimination in learning agents
Source: Proc Natl Acad Sci U S A. 2025 Jun 16;122(25):e2319933121. doi: 10.1073/pnas.2319933121 (PMC12207482; doi:10.1073/pnas.2319933121)
Supplement: Supplementary file 1 — Appendix 01 (PDF) [file pnas.2319933121.sapp.pdf]

Supporting Information: Perceptual interventions ameliorate statistical discrimination in learning agents

1 Environment details

The *boat race* environment is built using DMLab2D [2], which is a configurable and performant library for creating multi-agent 2D environments. The environment is open sourced as part of the Melting Pot 2.0 release [1]. The specific configuration used for the experiments in this article can be found at:

`https://github.com/google-deepmind/statistical_discrimination`

The environment consists of objects and avatars, where each avatar is controlled by a single agent, and the avatars can be used to interact with objects. We will refer to an agent controlling an avatar as a player, and we will refer to a player's actions or observations as they pertain to their avatar.

An episode on the environment consists of a number of races k, with $k \in \{2, 8\}$. Each race is separated in 3 phases, a partner choice phase lasting 65 steps, a semaphore changing phase lasting 5 steps, and a rowing phase lasting 230 steps. Each race runs in the opposite direction of the previous one, alternating North and South directions.

The objects in the environment are:

- **Apples**. Players entering the same location as the apple consume the apple and receive a reward. The apple will respawn depending on its location: at the end of a race for apples above the river; and with a probability of 10% per step for the apples on the goal river bank.
- **Barriers**. Barriers prevent players from entering their location, and are used to gate access to the boats before the rowing phase of the race starts.
- **Semaphores**. These are impassable objects that show a traffic-light pattern signaling the transition from the partner choice phase to the rowing phase of a race. They are colored red during partner choice, and change to yellow for the 5 steps of the semaphore changing phase, and then to green in the rowing phase of the race.
- **Boat**. Boats have two seats that players can enter. Once a player enters a seat, they control the oar next to it with their rowing actions. Players in a seat cannot move anymore, and all of their movement actions are no-ops. Boats can only move during the rowing phase of a race, and require two players, one on each seat to move. When a boat reaches the opposite river bank, it automatically disembarks its players into the river bank, past the barriers.
- **Water.** Water is impassable by players on foot (i.e. not in a boat). Boats with players can travel across water.

Episodes start with boats on the South river bank, with no apples on that bank, and its barriers closed. The North bank contains apples and its barriers are open. Once the rowing phase starts, the barriers toggle with the North ones being blocked and the South ones being open. At the beginning of the next race, apples on river banks also toggle, with the South ones appearing, and the North ones disappearing.

Players have movement actions and rowing actions. The players can move in any of the cardinal directions (North, East, South or West), and can also turn 90 degrees left or right. There are two rowing actions: paddle and flail. Rowing actions are considered no-ops when the players are not on a boat seat. Similarly, movement actions are no-ops when players are on a seat. Flailing actions can be executed at every time step, and have a 10% probability (non-cumulative for each player) of moving the boat forward at each time step. Paddling actions have a cool down of 2 steps, meaning the can only be executed every 3 steps. Paddling during the cool down period are considered no-ops. Paddling by a partner during the cool down phase will result in the boat moving 1 cell. Flailing by a partner during the cool down phase results in a reward punishment of -0.5 and the paddling attempt is wasted, and progress can only be made by flailing until the cool down ends.

Players have partial observability of 11×11 cells around them, with 5 cell to each side of their position, 1 cell behind, and 9 in front. They perceive their environment as RGB images of size $176 \times 176 \times 3$ (sprites are 16×16). Players who do not reach the other side of the river by the end of the race are disqualified and removed from the episode. The agent's observations in this case are fully black (zeros) and all their actions are no-ops.

Agents have a persistent color across episodes, which is instantiated as an avatar of that color in the level. Avatars can be of two colors: purple (base RGB = $(145, 30, 180)$) and teal (base RGB = $(30, 180, 145)$). In addition, the avatar is assigned a random badge for each episode, used to uniquely identify the players, but only within that episode. The badge is a set of 4 pixels that can be either black or white. The color of the agent is more salient than the badge (see FigureS1).

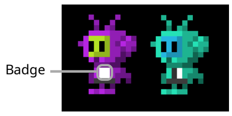

Fig. S1: The avatars in the boat race environment. We show avatars of each color facing left. The badge of the purple agent is all white, while the badge of the teal agent is half black and half white.

Apples on the river banks respawned at a rate of 0.1 per timestep, and confer a reward of +1 to the player who eats them.

The video youtu.be/nYbiQyT5Rxs shows a focal individual in an episode. To improve interpretability, we post-process the video to highlight the focal individual in white, cooperator bots in blue, and defector bots in red.

1.1 Environment validation

When the players are on the boat, they can choose from two different rowing actions at each timestamp: *paddle*, that is efficient, but costly if not coordinated with its partner; and *flail*, that is inefficient, but which effectiveness isn't affected by the partner's rowing style. When both players *paddle*, the boat moves one cell every 3 timesteps. When either player *flails*, the boat has a 10% probability of moving one cell, and a reward penalty of -0.5 is given to its partner if that partner is currently *paddling* (i.e. if they have executed the paddle action within the last 3 timesteps).

To verify that our environment has the properties of an iterated *Stag Hunt*, we compute in Figure S2 its *Schelling diagram* [9, 8]. A Schelling diagram is a plot that summarizes the incentives of a set of players who face a binary choice of a strategy to pursue. In our case the choice of strategy is either always paddle (paddler), or always flail (flailer). It is possible to read off various game theoretic properties from a Schelling diagram including whether a game is a social dilemma. The way to interpret the Schelling diagram is to consider a focal individual joining an episode where there are a certain number of cooperators (paddlers), and the rest are defectors (flailers) for a total of 5 other players. This focal individual then faces a binary choice of whether to join as a paddler, and receive the payoff that paddlers receive given the group composition, or receive the payoff of flailers. For instance, if the episode has 3 paddlers, and 2 flailers already, the focal player joining as a paddler would receive the payoff of the paddler's line at $x = 3$ in Figure S2 (on average ≈ 800 reward), and if joining as a flailer would receive the payoff of the flailer's line at $x = 3$ (on average ≈ 490). Therefore, if there are 3 paddlers already in the environment, there is an incentive to join as a paddler.

All agents trained are ACB agents as described in [1] (more details below). To obtain paddlers and flailers, we agents with a preferred rowing type. We give a pseudo-reward of +5 for executing their preferred rowing type, and of -5 for executing the wrong rowing type. All other rewards are left intact. We validated that paddlers overwhelmingly "paddle" when in the boat, while flailers "flail" (see below).

From Figure S2, we can see that when paddlers are abundant, paddling is advantageous, however, when paddlers are rare, flailing is advantageous, which correspond to a population version of *Stag Hunt* (as per the definition

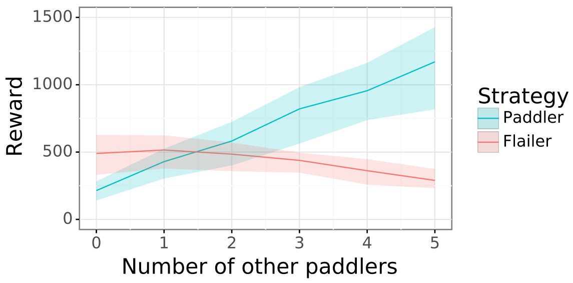

Fig. S2: The Schelling diagram of the *boat race* environment with 8 races ($k = 8$), unconditional cooperation (paddler), and defection (flailer). Depicted are the expected payoffs (average in solid line, quartiles in shaded area) that an individual joining an episode with a particular number of paddlers (on the x-axis) would obtain, depending on their choice of strategy (paddler or flailer).

proposed in [5]). Thus, we refer to paddlers as *cooperators* and flailers as *defectors*.

2 Training of community agents

To create the communities, we train and freeze agents to unconditionally cooperate or defect by directly incentivizing them with pseudo-rewards. A training community consists of 20 bots: n purple cooperators, n teal defectors, $10 - n$ purple defectors and $10 - n$ teal cooperators, for $n = 0, \ldots, 10$. Therefore, there are 11 possible community compositions. This way, a training community will always have 10 cooperators (and defectors), and 10 purple (teal) bots. What changes between compositions is the statistical association of color with strategy, and we refer to this as the *training bias*. Focal individuals will be assigned to a particular community, and train in episodes with 5 bots sampled (without replacement) from their training community.

The crown is shown depending on a value that tracks the rowing each player perform during the interaction phase. We refer to this value as the "crown value" χ. The crown value starts at 0 in the partner choice phase. Every time a player paddles its crown value is updated by $\chi \leftarrow 0.2 + 0.8 \cdot \chi$. Every timestep, regardless of whether the player is rowing or not, the crown value is decayed with parameter β as $\chi \leftarrow (1 - \beta) \cdot \chi$. When χ exceeds 0.6 the crown is shown, when it drops below 0.3, the crown is hidden.

We train focal individuals using ACB (actor-critic baseline) described in Melting Pot [1, 3]. We train one focal individual of each color for each of the 11 possible community compositions. Focal agents are independently trained for the baseline, the no memory agent, and for each level of salience of perceptual intervention: a decay (β) of the crown of $0.02, 0.005, 0.002, 0.0$. Focal individuals have no intrinsic preference for either type of rowing.

3 Agent details

All agent architectures had the same size convolutional net with two layers with output channels 16 and 32, with a stride of 8 pixels. The convolutional net was followed by a feedforward net with two layers, both with 64 output units. Agents with LSTM, have a hidden state of size 128, using an unroll length of 100. Agents were trained on mini-batches of data of size 16. Agents have a pop-art layer [4] for normalizing the value function. We also minimized a contrastive predictive coding loss [7] in the manner of an auxiliary objective [6], which in this case contrasted between nearby time points via LSTM state representations (a standard augmentation in recent work with A3C).

Agents for the training community and the production of the Schelling diagram were trained for 3×10^8 steps, whereas naive learners were trained for 1.5×10^9 steps. We used a learning rate of 4×10^{-4}.

Agents for the training community and Schelling diagram were incentivized to either unconditionally paddle or unconditionally flail. These agents receive a reward of $+5$ for every rowing that matches their incentive, and -5 if it didn't. Agents overwhelmingly learned to use their incentivized rowing. Across 350 episodes, paddlers never flailed in either 2 races or 8 races. Flailers paddled only twice out of over $200,000$ flailing actions, and only in the case of 2 races. Community bots did not exhibit any significant preference towards a particular boat, or a particular side seat within the boat (see Figure S3).

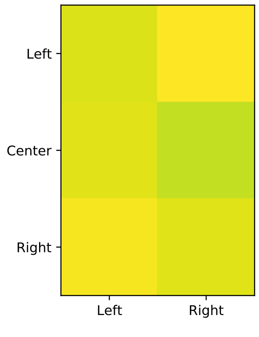

Fig. S3: Physical association of community bots who participated in a race. The figure shows a heat map of the counts for each time a bot was on each of the boats (left, center, right) taking one of the seats (left, right). The values ranged from 220 (darker), to 232 (lighter).

Out of $2,200$ episodes used for evaluation for each architecture, we counted the number of times the naive learner paddled and flailed (see Table T1). Given that paddling has a cool down, flailing would be expected to be 3 times as frequent if agents were rowing at random. Naive learners primarily learned to paddle, with the weakest case being

the ACB agent with LSTM, where the absolute flailing was greater than the absolute paddling, but only by a factor of two. We also calculated the correlation of the paddling and flailing on the first race of the naive learner with its partner. Overall, there was a positive correlation between the flailing for agents without an LSTM. The rest of the correlations were low (see Table T1).

Architecture	# flail	# paddle	flail corr.	paddle corr.
ACB LSTM	34,062	17,604	0.07	-0.12
ACB no LSTM	19,525	31,217	0.26	0.03

Table T1: Counts of the number of paddle actions and the number of flail actions for naive learners as well as the correlation between their rowing and that of their partner. All counts correspond to the first race for a total of $2,200$ episodes where the partners were uniformly random across strategy and color.

4 Discrimination index

We define the *association matrix* P of a naive learner as a 2×2 matrix of counts where $P_{i,j}$ corresponds to the number of times the naive learner shared a boat with a bot with color i and strategy j. We define the *participation* of an agent as the sum of the entries in their association matrix P. We define the *discrimination index* of a naive learner as

$$D = |P_{p,c} - P_{t,c}| + |P_{p,d} - P_{t,d}| - |P_{p,c} - P_{p,d}| - |P_{t,c} - P_{t,d}|$$

For simplicity, let's rename the entries of P as a, b, c, and d. Without loss of generality, $a > b, c, d$, thus there are 4 cases: $c > d \& b > d$, $c > d \& b < d$, $c < d \& b > d$, or $c < d \& b < d$. Note that $D = 0 \iff a > d > b, c$. Otherwise $D = 2(c - b)$ or $2(c - d)$ or $2(d - b)$ which is always even.

We simulated an individual sampling partners according to a particular strategy. Figures S4 and S5 show the histogram over $10,000$ simulations of an individual sampling uniformly at random, or sampling only based on one color, when present. We assume that the other 5 players are of a random color and random strategy, and that the focal individual always has first choice of partner.

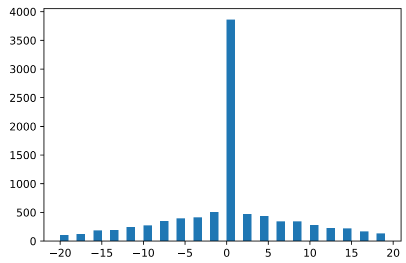

Fig. S4: Histogram of discrimination index for $10,000$ simulations of unbiased random sampling of partners.

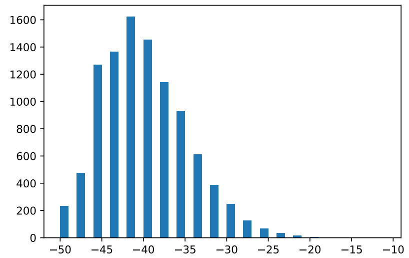

Fig. S5: Histogram of discrimination index for $10,000$ simulations of sampling cooperators (uniformly random), if any are available, otherwise, sampling a random partner.

References

[1] J. P. Agapiou, A. S. Vezhnevets, E. A. Duéñez-Guzmán, J. Matyas, Y. Mao, P. Sunehag, R. Köster, U. Madhushani, K. Kopparapu, R. Comanescu, et al. Melting pot 2.0. *arXiv preprint arXiv:2211.13746*, 2022.

[2] C. Beattie, T. Köppe, E. A. Duéñez-Guzmán, and J. Z. Leibo. Deepmind lab2d. *arXiv preprint arXiv:2011.07027*, 2020.

[3] L. Espeholt, H. Soyer, R. Munos, K. Simonyan, V. Mnih, T. Ward, Y. Doron, V. Firoiu, T. Harley, I. Dunning, et al. Impala: Scalable distributed deep-rl with importance weighted actor-learner architectures. In *International Conference on Machine Learning*, pages 1407–1416. PMLR, 2018.

[4] M. Hessel, H. Soyer, L. Espeholt, W. Czarnecki, S. Schmitt, and H. van Hasselt. Multi-task deep reinforcement learning with popart. In *Proceedings of the AAAI Conference on Artificial Intelligence*, volume 33(01), pages 3796–3803, 2019.

[5] E. Hughes, J. Z. Leibo, M. G. Phillips, K. Tuyls, E. A. Duéñez-Guzmán, A. García Castañeda, I. Dunning, T. Zhu, K. R. McKee, and R. e. a. Koster. Inequity aversion improves cooperation in intertemporal social dilemmas. *arXiv preprint arXiv:1803.08884*, 2018.

[6] M. Jaderberg, V. Mnih, W. M. Czarnecki, T. Schaul, J. Z. Leibo, D. Silver, and K. Kavukcuoglu. Reinforcement learning with unsupervised auxiliary tasks. *arXiv preprint arXiv:1611.05397*, 2016.

[7] A. v. d. Oord, Y. Li, and O. Vinyals. Representation learning with contrastive predictive coding. *arXiv preprint arXiv:1807.03748*, 2018.

[8] J. Pérolat, J. Z. Leibo, V. F. Zambaldi, C. Beattie, K. Tuyls, and T. Graepel. A multi-agent reinforcement learning model of common-pool resource appropriation. In *Advances in Neural Information Processing Systems 30: Annual Conference on Neural Information Processing Systems 2017, December 4-9, 2017, Long Beach, CA, USA*, pages 3643–3652, 2017.

[9] T. C. Schelling. Hockey helmets, concealed weapons, and daylight saving: A study of binary choices with externalities. *Journal of Conflict resolution*, 17(3):381–428, 1973.
